# Assessing the impact of psychological support on healthcare workers in conflict zones: a 5-Session intervention in North Kivu and Ituri

**DOI:** 10.1192/j.eurpsy.2025.543

**Published:** 2025-08-26

**Authors:** E. Dozio, V. Wamba

**Affiliations:** 1 Mental Health and Psychosocial Support, Action contre la Faim, Paris, France; 2 Mental Health and Psychosocial Support, Action contre la Faim, Goma, Congo, The Democratic Republic of the

## Abstract

**Introduction:**

North Kivu and Ituri in the Democratic Republic of the Congo (DRC) are among the provinces most severely affected by humanitarian needs. The security situation is highly unstable, with over 530,000 people displaced. Those remaining face urgent needs, and the conflict has severely impacted their mental health. An assessment of healthcare staff in local centers revealed significant distress due to both their exposure to patients in crisis and the surrounding violence and insecurity.

**Objectives:**

The aim of the protocol developed and proposed to healthcare staff was to implement preventive and curative phycological support actions designed to address all the psychosocial risks identified, as well as to alleviate emotional distress, improve well-being and strengthen the resilience mechanisms of healthcare centre staff.

**Methods:**

A 5-session protocol was proposed to healthcare staff. The sessions focused on psychological distress specific to professional situations in the context of conflict and humanitarian emergencies: vicarious trauma and self-help strategies using emotional regulation exercises. We measured several dimensions of participants’ psychological and professional well-being at the start and end of the programme: the PCL-5 to assess post-traumatic stress symptoms, the HADS to measure levels of anxiety and depression, the Maslach Burnout Inventory (MBI) to assess three dimensions of burnout: emotional exhaustion, depersonalisation and personal fulfilment. Finally, the ProQoL scale was used to assess participants’ quality of working life.

**Results:**

Analyses of the scores differences between pre and post intervetion, among a pre-sample of 65 participants (21% women; 78.5% men) showed significant improvements. Anxiety and depression levels decreased significantly (HAD-A: t = - t = 7.71, p < 0.001; HAD-D: t = 7.30, p < 0.001). On the MBI, participants showed a significant reduction in emotional exhaustion (t = 5.83, p < 0.001) and depersonalisation (t = 8.85, p < 0.001) and an increase in the sense of personal accomplishment (t = -5.12, p < 0.001). The results also show a clear reduction in post-traumatic stress symptoms (PCL-5: t = 8.64, p < 0.001). On the ProQoL scale, compassion satisfaction also increased significantly (t = -5.70, p < 0.001), indicating that carers feel more gratified by their role despite the challenges they face. Secondary traumatic stress (t = 5.38, p < 0.001), and burnout (t = 2.82, p = 0.006)., although significantly reduced, remain areas of concern.

**Conclusions:**

The intervention had significant positive effects on several dimensions of the psychological and professional well-being of healthcare workers, contributing to better stress management, increased satisfaction, and reduced burnout. These results highlight the importance of implementing support programs for healthcare professionals working in challenging contexts.

**Disclosure of Interest:**

None Declared

